# Cognitive Reappraisal Moderates the Longitudinal Relationship between Adolescents’ Peer Victimization and Self-Esteem. A Latent Interaction Model

**DOI:** 10.1007/s10578-024-01688-0

**Published:** 2024-03-06

**Authors:** Elli Spyropoulou, Theodoros Giovazolias

**Affiliations:** Department of Psychology, University of Crete, Gallos Campus, Rethymno, Crete 74150 Greece

**Keywords:** Self-esteem, Peer victimization, Cognitive reappraisal, Longitudinal, Latent interaction model, Adolescence

## Abstract

Poor self-esteem relates closely to youth maladjustment and appears to be predicted by peer victimization experiences. However, not all peer victimized adolescents face the same risk for self-esteem erosion over time. Drawing upon the Bi-Dimensional Framework for resilience and extant research, the present study examined the potential moderating role of cognitive reappraisal in the prospective relationship from peer victimization to self-esteem. To increase precision of findings the long-term impact of self-esteem on peer victimization was also tested. Self-reported data were collected from 285 early adolescents (*M*_age_ = 10.53 years, *SD* = 0.16; 54.0% girls) at two waves, spaced 1-year. Latent moderated structural equation analysis showed that peer victimization was negatively related to later self-esteem, but only for youth displaying low levels of cognitive reappraisal. For adolescents with high levels of cognitive reappraising, peer victimization was not found to predict any changes in self-esteem over time. The long-term impact of self-esteem on peer victimization was not supported. Overall the present study suggests that enhancing cognitive reappraisal could be a promising avenue for lowering risk for poor self-esteem in young individuals experiencing peer victimization.

## Introduction

Poor self-esteem relates closely to youth overall maladjustment, and appears to be predicted by prior exposure to peer victimization experiences [e.g.,^67^]. However, not all peer victimized adolescents face the same likelihood for self-esteem erosion over time [36, 67, 68]. Insight into factors that might buffer the negative long-term effects of peer victimization on young individuals’ self-esteem seems important both at theoretical and intervention level. Relevant literature has consistently shown that youth who can manage effectively emotions in response to interpersonal stressors are more likely to show resilience and achieve better life outcomes [e.g.,^19^]. Cognitive reappraisal (i.e., reinterpreting the meaning of a stimulus to alter its emotional impact) [32] is an emotion regulation strategy that has been strongly supported to protect young victims from aversive consequences [e.g.,^22^]. Surprisingly, whether cognitive reappraisal buffers the negative relationship between peer victimization and later self-esteem in youth has not been addressed in any published article yet. Drawing upon the Bi-Dimensional Framework for resilience [45] and extant research, the present study aimed to examine the potential moderating role of cognitive reappraisal in the prospective relationship between peer victimization and self-esteem across early adolescence. To increase precision of findings the long-term impact of self-esteem on peer victimization was also tested.

Self-esteem represents the evaluative and affective component of self-concept, with the latter describing individuals’ perceptions of their abilities in different life domains [40]. In other words, self-esteem denotes how much individuals like themselves, how much they feel pleased with themselves and the way they function in life [40]. The importance of self-esteem on psychological health and subjective well-being has long been emphasized by Branden who stated that “self-esteem has profound consequences for every aspect of our existence” (1994, p.5). In line with this notion, longitudinal research has consistently shown that individuals possessing high levels of self-esteem are more likely to display increased levels of optimism [41], life satisfaction [57] as well as adaptation to stressful life events [25]. They also tend to experience positive interpersonal relationships [38], and usually show greater academic achievement [94]. Conversely, low self-esteem has been prospectively linked with poor health outcomes, including anxiety, depression [78], suicidal ideation [60] and substance dependence [8]. Self-esteem represents a core aspect of adolescents’ psychological functioning with boys usually reporting higher levels of self-esteem than girls [e.g.,^1^]. Literature findings strongly indicate self-esteem to be low in consistency across early adolescence and to become relative stable as individuals grow older [5, 82]. This is important to consider as a low in stability self can be more amenable to change and, thus, more likely to be defined and influenced by a broad range of factors [7, 74], including interpersonal ones [65].

Peer victimization is a widespread interpersonal problem that appears to be closely associated with youth’s self-esteem both concurrently and over-time [9, 66, 67, 83]. Being usually experienced more frequently by boys than girls [77], peer victimization can lead to negative feelings for the self as it conveys negative information to the victim which may, in turn, be internalized [72]. Particularly, perceived devaluation by peers is likely to damage self-esteem by ensuing feelings of shame [13, 44, 92] and incompetence regarding one’s own ability to function socially [54, 71]. Although poor self-esteem can be a consequence of peer victimization, youth with poor self-esteem can also trigger harassment by peers, and, thus, be trapped in a harmful vicious cycle that is hard to escape from [85]. Identifying factors that might protect young victims from self-esteem erosion over time could break this cycle and reduce likelihood for further peer abuse and later adjustment difficulties. Empirical findings strongly support that emotion regulation in response to life stressors can help youth become resilient and experience better life outcomes [19, 75].

One widely studied form of emotion regulation is cognitive reappraisal, an antecedent-focused strategy that intervenes before the activation of an emotional response [32]. Being effectively employed by children as young as six years old [91], cognitive reappraisal is mostly regarded as an adaptive strategy that involves changing the meaning or self-relevance of an event to alter its emotional impact [32]. Reappraising recognizes that emotional responses represent an outcome of one’s own thoughts or appraisals [61]. Hence, the same external experience can activate pleasant or unpleasant emotions based on how it is perceived and interpreted by individuals. Being usually equally endorsed by both sexes in adolescence [35] cognitive reappraisal has been related with positive adjustment outcomes, including better interpersonal functioning [e.g., 3], positive affectivity [88] and engagement in problem solving [81]. Habitual use of cognitive reappraisal (a person’s disposition in using cognitive reappraisal in contrast of being instructed to do so) can also help individuals to hold a positive attitude toward the self and to accept both good and bad qualities [6, 31].

## Cognitive Reappraisal as a Resilience Factor

According to the Bi-Dimensional Framework for resilience [BDF; 45] resilience factors represent those internal characteristics that act to attenuate the impact of risk upon the development of aversive outcomes [45]. Unlike other resilience approaches, the BDF does not focus upon identifying “positive” factors that are inversely related with aversive outcomes [e.g., 4]. Instead, BDF’s emphasis is on detecting those psychological factors that interact with or statistically moderate the likelihood that risk will lead to negative consequences [46].

Cognitive reappraisal as an inner-state process that protects young victims from maladjustment outcomes has been established both concurrently and over time. Particularly, in a cross-sectional study conducted with 582 adolescents, engagement in cognitive reappraisal was found to reduce likelihood for anxiety associated with peer victimization [29]. Similarly, cognitive reappraisal alleviated symptoms of depression in 338 young victims [22], and dampened the association between stressful life events and suicide ideation in 175 depressed adolescent girls [26]. Accordingly, longitudinal research showed cognitive reappraisal to mitigate risk for depressive symptoms following peer victimization among 1,823 young individuals [95] as well as to reduce likelihood for mental health problems in cybervictims [84].

Cognitive reappraisal entails changing the meaning of a situation or intentionally focusing on its’ positive aspects to alter its emotional impact [32, 33]. Young victims who are able to engage in cognitive reappraisal may consider alternative interpretations of peers’ provocations, instead of taking them personally, thus increasing the generation of negative self-evaluations (e.g., “my peers bother me because they feel lonely and want to attract attention”). They can also view victimization by peers as a positive challenge rather than a threatening and provocative experience, and, thus, manage stress effectively, and feel better for themselves. For instance, a young victim who can reappraise peer victimization as an opportunity to stand up for oneself and seek social support may experience positive feelings for the self (e.g., pride) instead of shame. Surprisingly, whether cognitive reappraisal protects against self-esteem erosion in peer victimized adolescents has not been addressed in any published article yet.

## The Current Study

Despite of evidence showing that cognitive reappraisal can reduce the negative relationship between peer victimization and later self-esteem in adolescence, this possibility has not been examined yet. Drawing upon the Bi-Dimensional Framework for resilience and extant research, the present study aimed to examine the potential moderating role of cognitive reappraisal in the prospective relationship between peer victimization and self-esteem across early adolescence. To increase precision of findings the long-term impact of self-esteem on peer victimization was also tested. It was expected that cognitive reappraisal would moderate the longitudinal relation between peer victimization and self-esteem, such that as cognitive reappraisal increased, the negative relationship between peer victimization and later self-esteem would become attenuated (Hypothesis 1). Building upon prior findings self-esteem was expected to be negatively associated with peer victimization over time (Hypothesis 2).

## Methods

### Participants and Procedure

Convenience sampling method was employed to recruit participants from 13 public primary schools, all located in the prefecture of Heraklion of Crete in Greece. A total of 24 classes (on average 2 classes per school) participated, with an average size of approximately 12 students per class. An enveloped letter, enclosing detailed information about the research procedure, parental consent form, as well as a brief demographic questionnaire (e.g. parents’ education) was initially sent to all parents/guardians of 5th graders in participating schools. In the Greek educational system, primary education lasts 6 years, and includes grades 1 to 6. Students with signed parental agreement were informed regarding the private, confidential, voluntary, and no compensatory nature of the study. Those who orally confirmed their participation (students’ oral assent was a prerequisite for data collection), received a personal code and, then, completed three paper-and-pencil self-report measures in small groups at school under the supervision of the researcher. The convenience sample at Time 1 (T1) comprised of 369 students (consent rate = 82%; *M*_age_ = 10.60, *SD* = 0.18; 54.29% girls). The time required to complete the study measures varied from 20 to 25 min. Participants were recontacted via their school for a follow-up, 1 year later [Time 2 (T2)], and were requested to participate again during class time. Data collection in both waves (T1/T2) took place approximately two months after the start of fall semester. Absenteeism at T2 (attrition rate = 18.2%) and validity checks (e.g., multivariate outliers) reduced the initial sample to 285 participants (*M*_age_ = 10.53 years, *SD* = 0.16; 54.0% girls.) which was considered satisfactory enough (> 200) for proceeding with structural equation modeling [SEM; 50]. No significant differences were found between students who participated at both temporal occasions and those who responded only at first occasion neither in sex, *χ*^2^_(1)_ = 1.07, *p* = 0.30 nor in the study variables [peer victimization, *t*_(363)_ = 1.70, *p* = 0.09; cognitive reappraisal, *t*_(363)_ = − 0.10, *p* = 0.92; self-esteem, *t*_(367)_ = − 0.62, *p* = 0.54]. Educational level of participants’ fathers varied: 14 (4.9%) had only elementary school education; 174 (61.1%) were high school graduates; and, 97 (34.03%) were university graduates. With regard to participants’ mothers education level: 4 (1.4%) had only elementary school education; 148 (51.9%) were high school graduates; and, 133 (46.7%) were university graduates. All participants were Greek ethnicity and Greek-language speaking. There were not any important changes in classrooms’ composition across both times of measurement. All procedures were approved by the Institute of Educational Policy and the Greek Ministry of Education.

### Data Collection Tools

#### Peer Victimization

Peer victimization was measured at T1 and T2 by the 9-item Victimization of Self subscale derived from the Peer Experiences Questionnaire-Standard Version [PEQ-STDV; 30, 86]. Respondents were asked to indicate on a 5-point Likert scale (1 = never to 5 = few times a week) the frequency of experiencing peer victimization in the school context the last three months (e.g., “*A student lied about me in order for the other students not to like me*”). Internal consistency of the Victimization of Self subscale was (T1, *α* = 0.84; T2, *α* = 0.83).

### Cognitive Reappraisal

Cognitive reappraisal was measured at T1 by the 6-item cognitive reappraisal subscale derived from the Emotion Regulation Questionnaire for Children and Adolescents [ERQ-CA; 35]. Respondents were asked to quantify on a 5-point Likert scale (1 = strongly disagree to 5 = strongly agree) the agreement to the behavior described (e.g., “*When I am worried about something, I make myself think about it in a way that helps me feel better*”). Internal consistency of the Cognitive Reappraisal subscale was (T1, *α* = 0.75).

### Self-Esteem

Self-Esteem was measured at T1 and T2 by the 5-item global self-esteem subscale derived from the Greek version of the Self-Perception Profile for Children [SPPC; 39, 55]. In SPPC each item is presented by a binary statement (e.g., “*Some kids are satisfied with themselves*” and “*Other kids are not satisfied with themselves*”). Participants were asked to decide which statement describes them best and to choose accordingly the “Really true for me” or “Sort of true for me” option. In SPPC answers are scored on a 4-point Likert scale from 1 to 4 with higher scores indicating greater levels of self-esteem. For example, the “Really true for me” choice gives a score of 4 to the statement “Some kids are satisfied with themselves” and a score of 1 to the statement “Other kids are not satisfied with themselves”. Accordingly, the “Sort of true for me” choice gives a score of 3 to the statement “Some kids are satisfied with themselves” and a score of 2 to the statement “Other kids are not satisfied with themselves”. Internal consistency of the Self-Esteem subscale was (T1, *α* = 0.73; T2, *α* = 0.78).

### Control Variables

Sex assigned at birth was self-reported and coded as “0 = boys” and “1 = girls”.

### Analysis Strategy

Statistical analyses were conducted using IBM Statistical Package for the Social Science (SPSS) version 25.0 and the Mplus version 8.1 [64]. Univariate normality was not violated as skewness and kurtosis were within the proposed ranges of ± 2 and ± 10 [48]; (see Table 1). Multivariate outliers were evaluated using Mahalanobis distance (*p* < 0.001) and treated by removal [49] Common-method bias did not seem to pose any serious challenge on study’s conclusions, as the Harman’s single factor test indicated a first common factor with variance │16.47%│ which was less than the critical criteria of 40% [70]. Bivariate associations between variables were tested using Pearson correlations (*r* = 0.10 to 0.29, small; *r* = 0.30 to 0.50, medium; *r* > 0.50, large) [18]. Independent *t* tests were used to assess sex mean differences in all study variables. Missing data were at 0.14%. Little’s MCAR test was not significant, *χ*^2^_(385)_ = 417.89, *p* = 0.12, implying missing completely at random data. Full information maximum likelihood (FIML) with robust maximum likelihood estimator [MLR; 73] was, therefore, applied to ensure that all available piece of information was used, and to correct for significant departure from multivariate normality (skewness = 252.03; kurtosis = 347.54, *p* < 0.001) [56]. The self-esteem subscale was represented by its five indicators (T1/T2) whereas the six items of the cognitive reappraisal and the nine items of the peer victimization (T1/T2) subscales were randomly aggregated to form three parcels each [59].

**Table 1:** Descriptive statistics and bivariate correlations (Pearson’s r) for the study variables

Variables	1	2	3	4	5	Minimum	Maximum	Mean	SD	Skewness	Kurtosis
1. Peer Victimization T1	-					9	42	14.84	6.20	1.41	1.81
2. Peer Victimization T2	0.36**	-				9	45	13.77	5.75	2.05	6.02
3. Cognitive Reappraisal T1	− 0.02	− 0.04	-			6	30	22.33	4.39	− 0.65	0.78
4. Self-esteem T1	− 0.28**	− 0.19**	0.17**	-		7	20	16.50	2.75	− 0.79	0.44
5. Self-esteem T2	− 0.21**	− 0.35**	0.18**	0.37**	-	6	20	16.32	2.73	0.15	0.39

*Note*. *T1* = Time 1, *T2* = Time 2. **p < 0.01

Confirmatory factor analysis (CFA) was initially employed to test measurement fit of the hypothesized five-factor model [Cognitive Reappraisal (T1) + Peer Victimization (T1) + Peer Victimization (T2) + Self-Esteem (T1) + Self-Esteem (T2)], as well as to detect any possible estimation bias [58]. Constructs’ convergent validity would be established with standardized factor loadings being ≥ 0.32 [79] and statistically significant (*p* < 0.05). Accordingly, constructs’ discriminant validity would be accomplished with factor inter-correlations ≤ 0.80 [12]. Considering the sensitivity of the chi-square statistic (*χ*^2^) to the sample size, the *χ*^2^ to the respective degrees of freedom (*χ*^2^/df), the comparative fit index (CFI), the Tucker-Lewis index (TLI), and the root mean square error of approximation (RMSEA) were also used to evaluate models’ fit. A non-significant *χ*^2^ or alternative a ratio of ≤ 2 for *χ*^2^/df, and values of ≥ 0.90 for CFI/TLI, and ≤ 0.08 for RMSEA would constitute good fit [50].

After ensuring measurement fit of the hypothesized five-factor model, study hypotheses were tested with a two-step estimation procedure for estimating Latent Moderated Structural (LMS) equations [47] in Mplus vs. 8.1. LMS modeling is generally suggested to outperform the traditional composite score approach as it removes measurement error and, thus, produces less biased estimates of moderation effects. Particularly, the first step was to examine the structural model without the latent interaction (M0). The second step was to examine the structural model with latent interaction (M1) [for an application of this estimation procedure; 58]. M0’s data fit was assessed using the multiple indices and the typical interpretation guidelines described above [50]. Given that conventional model-data fit statistics have not been developed for LMS models, the log-likelihood ratio test denoted as D (D = -2[(log-likelihood for Model 0) – (log-likelihood form Model 1)]) was performed to compare the relative fit of M0 and M1. In Mplus output, the log-likelihood value used to perform this calculation is labeled “H0 Value” and is reported to outperform the robust difference test [73]. The significance of D value was assessed with the *χ*^2^ distribution. A significant D indicates that M1 (i.e., model with the interaction term) is also a well-fitted model and that M0 (i.e., model without the interaction term) represents a significant loss in fit relative to the more complex M1. A non-significant D shows that M0 does not result in a significant loss of fit relative to M1, and, thus, no conclusions regarding M1’s fit can be made (M1’s fit is equal to or worse than that of M0).

Using the model constraint command in Mplus vs. 8.1, cognitive reappraisal was divided into two levels: High level (1 standard deviation above the mean) and Low level (1 standard deviation below the mean). A significant interaction term would also be interpreted by graphing as in standard regression models [2]; in interaction graph regression coefficients for main effects and the latent interaction effect would be obtained from M1 [21]. To increase precision of findings, the cross-lagged path from self-esteem at T1 to peer victimization at T2, and sex as control variable were included in both M0 and M1. Sex-based differences were detected only in mean levels of self-esteem (T1/T2). Therefore, only self-esteem (T1/T2) was regressed on sex. Significance level was set to *p* < 0.05.

## Results

### Descriptive Statistics

Descriptive statistics and bivariate correlations for all study variables are presented in Table 1. All but one associations were significant, small to medium, and in the excepted direction. Significantly greater levels were found among boys than girls in self-esteem at both times of measurement (T1, *t*_(283)_ = 2.07, *p* = 0.04; T2, *t*_(281)_ = 2.08, *p* = 0.04). No significant sex-based differences were detected in mean levels of peer victimization (T1, *t*_(282)_ = − 0.84, *p* = 0.40; T2, *t*_(276)_ = − 0.27, *p* = 0.79) and cognitive reappraisal (T1, *t*_(298)_ = 1.47, *p* = 0.14).

### Measurement Model

The hypothesized five-factor model, *χ*^2^_(142)_ = 150.86, *p* = 0.29, *χ*^2^/df = 1.06, CFI = 0.99, TLI = 0.99, RMSEA [90%CI] = 0.02 [0.00, 0.03] showed very good fit to the data. Constructs’ validity was supported as all factor loadings were statistically significant, *p* < 0.001, and exceeded (*β*s = 0.52 to 0.82) the recommended cut-off value of 0.32 [79]. Constructs’ discriminant validity was also established as all factor inter-correlations (*r*s = 0.22 to 0.35, *p* < 0.05) were under the recommended cut-off value of 0.80 [12]. Overall, measurement properties of the hypothesized five-factor model were satisfied in order to proceed with testing the main and interaction effects.

### Main Effects Model

**Fig. 1:**
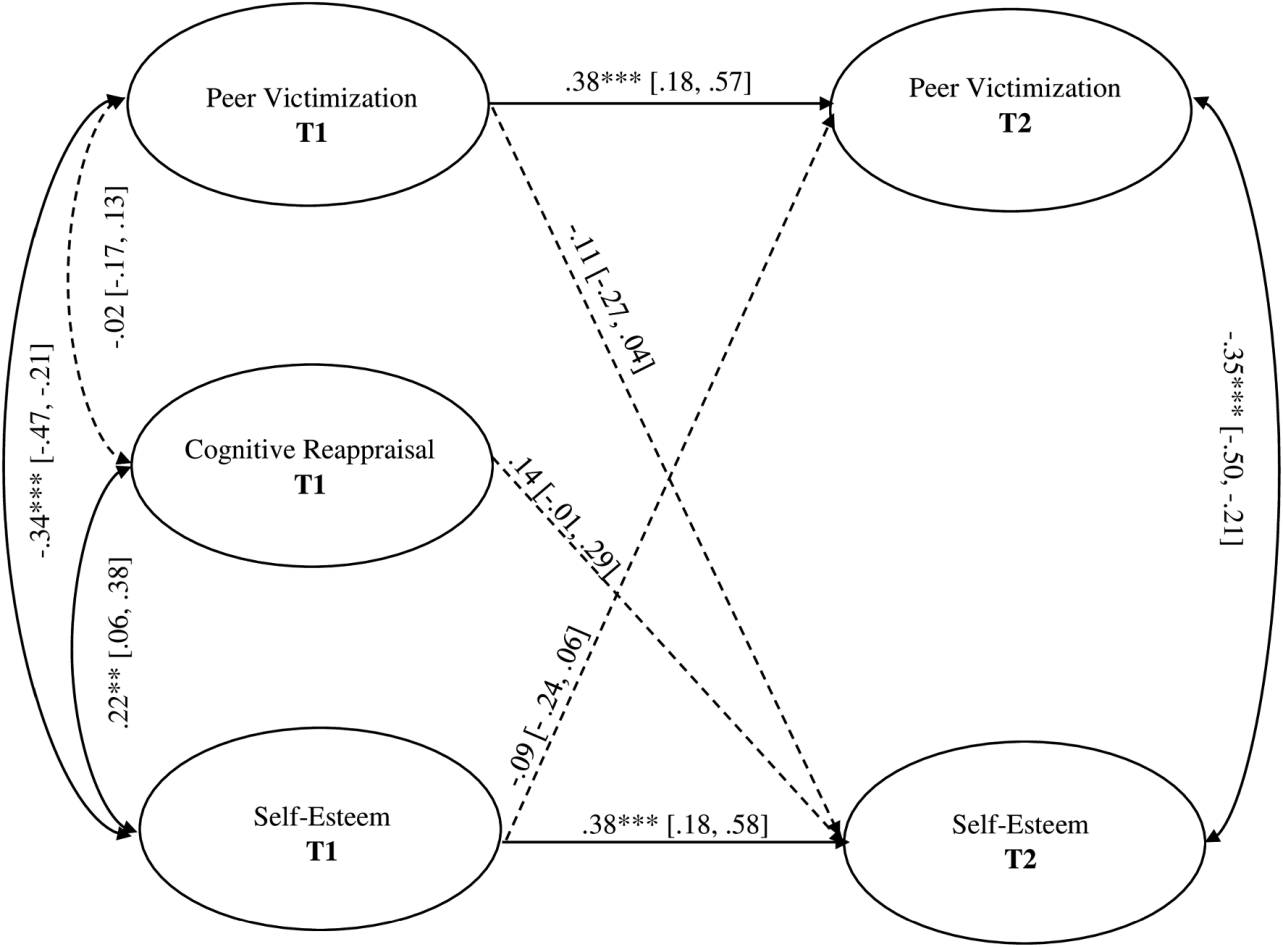
Model without latent variable interaction (M0). *Note.* Oval boxes indicate latent variables. Bidirectional arrows represent correlations between latent factors. Factor loadings and sex as a control variable (0 = boy 1 = girl) are not depicted for graphic simplicity. Values displayed are standardized. 95% confidence intervals are included in parentheses. Log-likelihood (H0) = - 8,654. **p < 0.01, ***p < 0.001

The main effects model (M0; Fig. 1) tested whether cognitive reappraisal at T1 and peer victimization at T1 uniquely predicted self-esteem at T2. The cross-lagged path from self-esteem at T1 to peer victimization at T2, and sex as control variable (self-esteem at T1/T2 was regressed on sex) were also included in the model. The full model explained 24% of the variance in self-esteem at T2, and fitted the data very well: *χ*^2^_(158)_ = 165.41, *p* = 0.32, *χ*^2^/df = 1.04, CFI = 0.99, TLI = 0.99, RMSEA [90%CI] = 0.01 [0.00, 0.03]. Cognitive reappraisal at T1 and peer victimization at T1 were not found to predict any changes in self-esteem at T2: cognitive reappraisal, *β* = 0.14, *SE* = 0.08, *p* = 0.06, 95% CI = − 0.01, 0.29; peer victimization, *β* = − 0.11, *SE* = 0.08, *p* = 0.15, 95% CI = − 0.27, 0.04. The statistical significance of the cross-lagged path from self-esteem at T1 to peer victimization at T2 was also not supported, *β* = − 0.09, *SE* = 0.08, *p* = 0.26, 95% CI = − 0.23, 0.07. Sex showed a significant effect only on self-esteem at T1, *β* = − 0.14, *SE* = 0.06, *p* < 0.05, 95% CI = − 0.27, − 0.01; T2, *β* = − 0.09, *SE* = 0.06, *p* = 0.15, 95% CI = − 0.21, 0.03.

### Latent Interaction Model

**Fig. 2:**
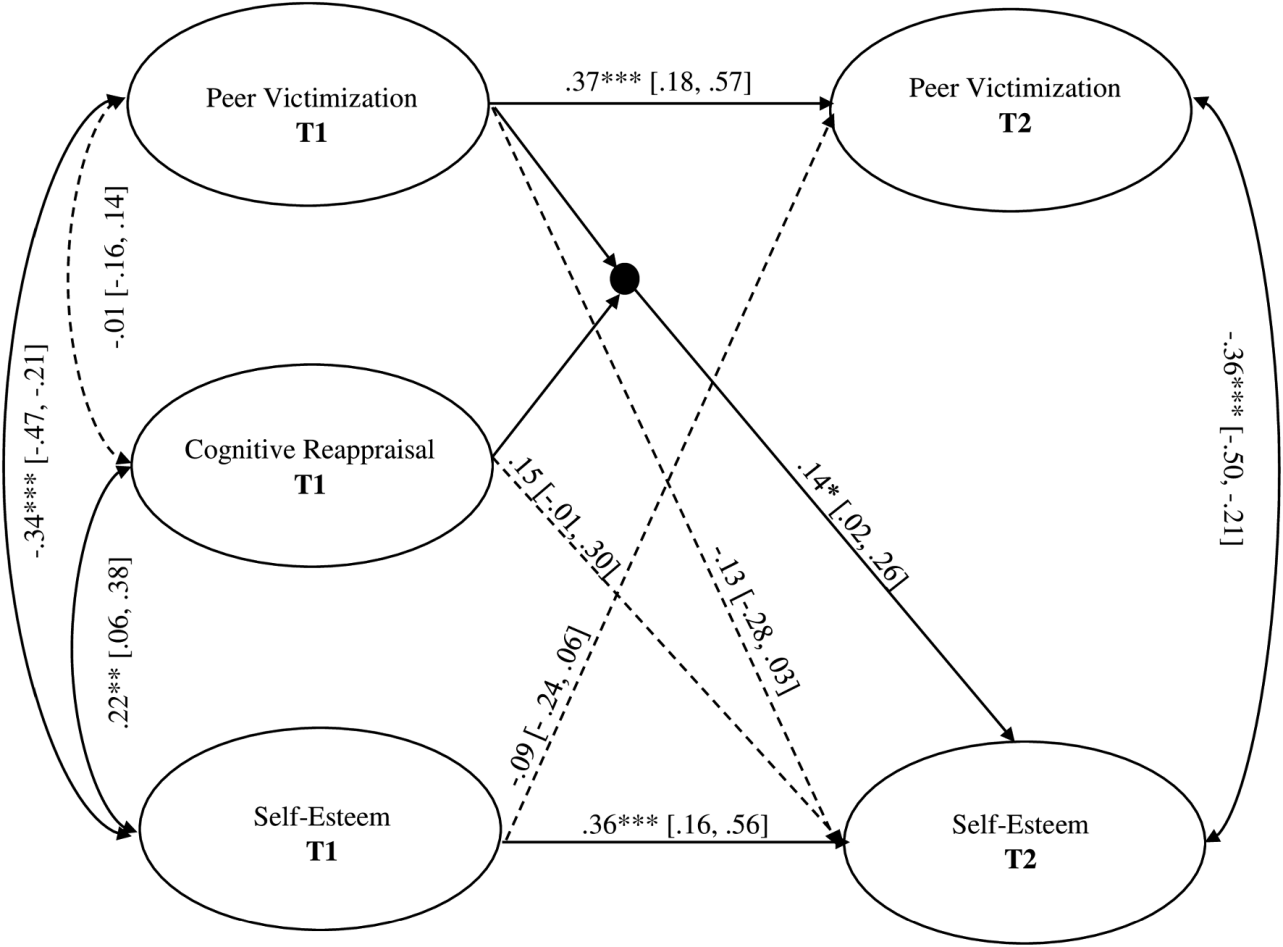
Model with latent variable interaction (M1). *Note.* Oval boxes indicate latent variables. Bidirectional arrows represent correlations between latent factors. Factor loadings and sex as a control variable (0 = boy 1 = girl) are not depicted for graphic simplicity. Values displayed are standardized. 95% confidence intervals are included in parentheses. Log-likelihood (H1)= - 8,652. *p < 0.05, **p < 0.01, ***p < 0.001

The interaction model (M1; Fig. 2) tested whether cognitive reappraisal at T1 and peer victimization at T1, and the interaction term (cognitive reappraisal at T1*peer victimization at T1) were all related to self-esteem at T2. Similarly to M0, the cross-lagged path from self-esteem at T1 to peer victimization at T2, and sex as control variable were also included in the model. The full model explained 26% of the variance in self-esteem at T2. Neither cognitive reappraisal at T1 nor peer victimization at T1 were related to self-esteem at T2: cognitive reappraisal, *β* = 0.15, *SE* = 0.08, *p* = 0.06, 95% CI = − 0.01, 0.30; peer victimization, *β* = − 0.13, *SE* = 0.08, *p* = 0.10, 95% CI = − 0.28, 0.03. The cross-lagged path from self-esteem at T1 to peer victimization at T2 was not statistically supported, *β* = − 0.09, *SE* = 0.08, *p* = 0.26, 95% CI = − 0.24, 0.06. Sex showed a significant effect only on self-esteem at T1, *β* = − 0.14, *SE* = 0.06, *p* < 0.05, 95% CI = − 0.26, − 0.01; T2, *β* = − 0.08, *SE* = 0.06, *p* = 0.15, 95% CI = − 0.19, 0.04.

**Fig. 3:**
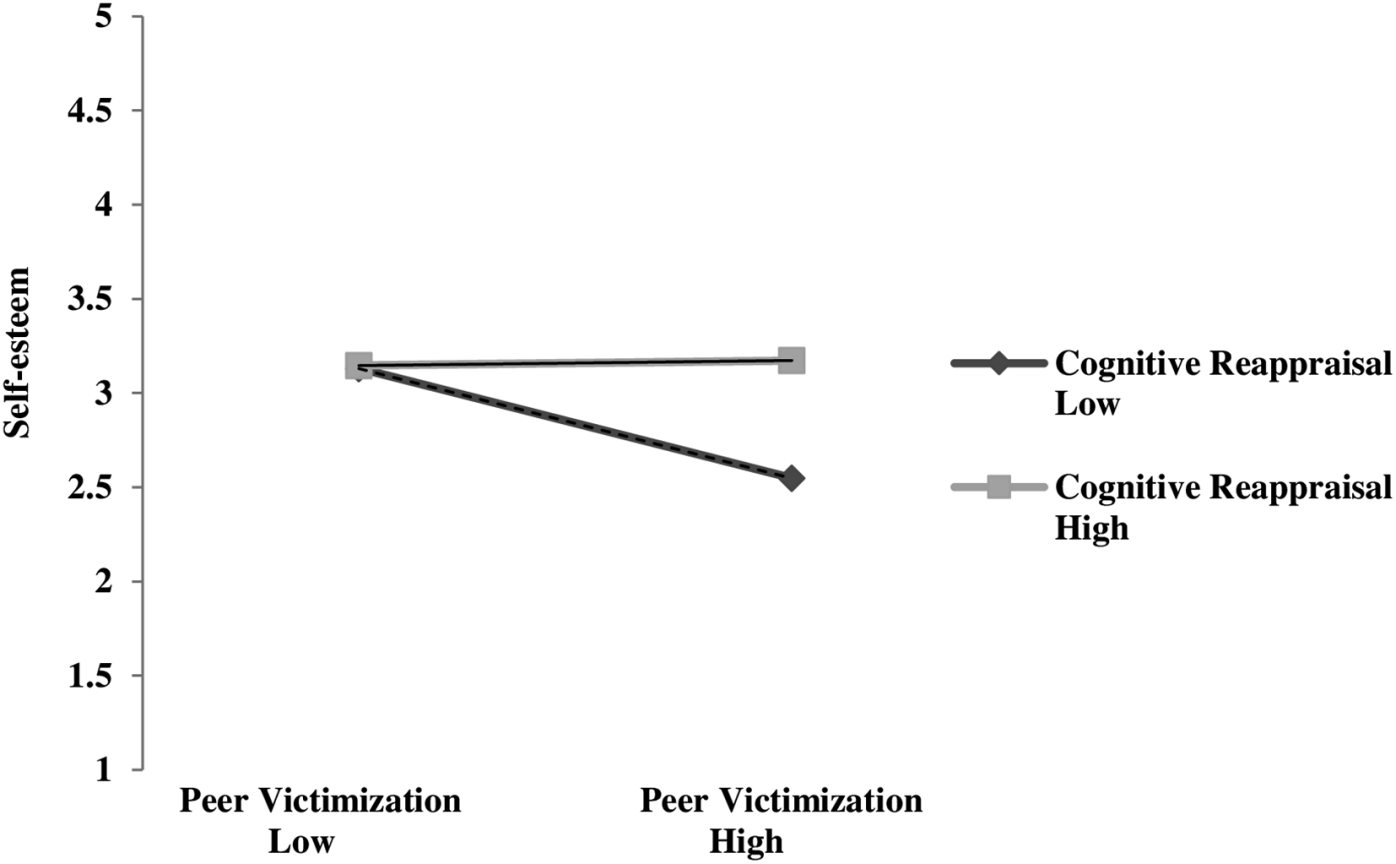
The interaction effect of peer victimization and cognitive reappraisal on later self-esteem. Low = 1SD below the mean. High = 1 SD above the mean

Using a *χ*^2^ distribution, the log-likelihood ratio test was not found to be significant (D = 3.9, *p* = 0.07), indicating that M0 (i.e., model without the interaction effect) did not represent a significant loss in fit relative to M1 (i.e., model with the interaction term). Therefore, no conclusions regarding M1’s fit could be made; M1 could be equal to or worse than M0. The interaction effect was significant, *β* = 0.14, *SE* = 0.06, *p* = 0.02, 95% CI = 0.02, 0.26. Peer victimization was negatively related to later self-esteem, but only for youth displaying low levels of cognitive reappraisal, *b* = 0.29, *SE* = 0.12, *p* < 0.05). For early adolescents with high levels of cognitive reappraisal, peer victimization was not found to predict any changes in self-esteem over time, *b* = 0.01, *SE* = 0.01, *p* = 0.89. Figure 3 represents the different relationship between peer victimization at T1 and self-esteem at T2 depending on the level of cognitive reappraisal at T1. The interaction plot showed that for early adolescents displaying high levels of cognitive reappraisal at T1 (1 standard deviation above the mean) the slope was relative stable in the prediction of peer victimization at T1 on self-esteem at T2, whereas for early adolescents displaying low levels of cognitive reappraisal (1 standard deviation below the mean), the slope was relative steep. This graph indicates that early adolescents with low levels of cognitive reappraisal are more vulnerable to low self-esteem following peer victimization, and that cognitive reappraisal mitigates the prospective effect of peer victimization on self-esteem.

## Discussion

Despite of evidence showing that low self-esteem can be predicted by prior peer victimization in adolescence, less is known about the conditions in which this relationship occurs. Guided by the Bi-Dimensional Framework for resilience and extant research, the present study aimed to examine the potential moderating role of cognitive reappraisal in the prospective relationship between peer victimization and later self-esteem among early adolescents. The long-term impact of self-esteem on peer victimization was also tested. Results showed that cognitive reappraisal served as a resilience factor by attenuating the negative effects of peer victimization on early adolescents’ self-esteem over time. Self-esteem was not found to predict any prospective changes on peer victimization.

As it was expected, cognitive reappraisal moderated the longitudinal relationship between peer victimization and self-esteem across adolescence. Particularly, earlier experiences of peer victimization were found to be negatively related to later self-esteem only for youth reporting low levels of cognitive reappraisal. In contrast, for early adolescents with high levels of cognitive reappraisal, peer victimization was not found to predict any long-term changes in self-esteem. The present findings seem to advance prior research indicating that using cognitive reappraisal in the context of peer victimization can be protective against maladjustment outcomes [e.g.,^95^]. In support of the Bi-Dimensional Framework for resilience [45], cognitive reappraisal seemed to reduce the likelihood that peer victimization will lead to lower self-esteem over time, thus acting as a resilience factor.

The finding that high levels of cognitive reappraisal buffered the risk for self-esteem erosion among young victims underscores the crucial role that cognitive reappraisal can serve in the development of a healthy self. Most importantly, it shows that it may not be peer victimization per se that deteriorates self-esteem, but rather the way young victims interpret this interpersonal experience [63]. For instance, a young victim who interprets peer victimization as an opportunity to stand up for oneself and seek social support may experience positive feelings for the self (e.g., pride) instead of shame. Indeed, evidence has shown cognitive reappraisal to be linked to feelings of authentic pride (i.e., one’s success is attributed to internal, unstable, and controllable events) and related self-worth [53]. Moreover, in an experimental study conducted with 113 students, instructed cognitive reappraisal was found to be beneficial in decreasing both state-shame and negative self-evaluations [15]. Young victims’ ability to control bad feelings by changing the way they think about them could also increase self-esteem by enhancing perceptions of self-efficacy [24]. In support of this notion, generalized self-efficacy as well as self-efficacy beliefs in one’s ability to manage negative emotions were found to be positively related to self-esteem over time [14, 52]. Experiencing the process of changing negative emotions and thoughts through reappraising can foster decentering, described as one’s ability to keep a distanced third-person perspective and non-judging stance towards distractive self-evaluative thoughts and emotions [51]. Decentering is shown to help people perceive emotions and thoughts as temporary mental events [51], and, thus, be less consumed by the thoughts and emotions that characterize low self-esteem [69, 90]. Finally, higher use of cognitive reappraisal could protect young victims from repetitive rumination thinking [16], and, thus, lower likelihood for low self-esteem over time [37].

In contrast with previous studies the long-term impact of self-esteem on peer victimization was not statistically supported [85]. In other words, self-esteem was not found to trigger any peer victimization experiences over time. The non-significant effects of self-esteem on later peer victimization could be accounted for by several reasons. For instance, although some adolescents may have lower self-esteem this may be high enough to maintain their position in the peer hierarchy, thus, decreasing risk for peer harassment [67]. The high variability that self-esteem usually displays across early adolescence [5] could be another reason for the non predictive effects of self-esteem on later peer victimization reported herein. Low self-esteem may be more likely to trigger peer victimization in later stages of development when it shows more stable and trait-like qualities [17]. However, it is also possible the prospective relationship between self-esteem and peer victimization to be condition-dependent. For instance, in a longitudinal three-wave study performed with 774 adolescents, low self-esteem was found to predict increases in peer victimization only for youth with a higher tendency to suppress their impulses, needs and feelings [67]. In a similar vein, low self-esteem increased likelihood for later victimization, only when adolescents perceived low levels of teacher support [36].

### Strengths, Limitations, and Future Directions

To our knowledge, the present study was the first to explore cognitive reappraisal as a potential moderator in the prospective relationship between peer victimization and self-esteem across early adolescence. Following previous recommendations [22] a longitudinal design was employed which overcomes methodological shortcomings related to cross-sectional research. Moderation hypothesis was examined with LMS modeling which outperforms the traditional composite score approach as it removes measurement error, and, thus, produces less biased estimates of moderation effects [47]. Building upon prior research the cross-lagged path from self-esteem to peer victimization was also controlled for.

Along with strengths, there are also some limitations that should be mentioned as they provide interesting directions for future research. In the present study only self-reports were used. Although common-method variance was not found to pose any threat on study’s validity, social desirability issues may have artificially masked true variable correlations [80]. Multi-informant (e.g., peer nominations) as well as multi-method approaches [e.g., fMRI for cognitive reappraisal; 62] could be employed so as to strengthen relevant research in the future.

Another caveat involves the non-stratified and non-clinical sample, which precludes generalization of findings. For instance, research documents victimization experiences to be more frequent among depressed individuals as compared to non-depressed ones [76]. Similarly, peer victimization related to ethnic group membership is shown to exert more negative influences on the self as compared to peer victimization related to personal characteristics [87]. Future research could, thus, benefit by testing the hypothesis tested herein in more diverse populations and clinical samples as well.

In the present study a general measure of cognitive reappraisal was employed which limits a more deep insight into the role that this emotion-regulation strategy could serve in the context of peer victimization. It would be important for future studies to test the current moderation hypothesis with measures of cognitive reappraisal specific to the context of peer victimization. Similarly, given that self-esteem may be hardly understood when only its global component is considered, domain-specific facets of self-esteem (e.g., relational) are suggested to be taken into account by relevant research in the future [89].

It would also be interesting for future studies to explore whether cognitive reappraisal lowers risk for self-esteem erosion in other types of interpersonal adversities, such as child maltreatment [e.g., emotional abuse, sexual abuse; 93]. For instance, higher engagement in cognitive reappraisal was found to buffer the negative effect of childhood emotional neglect on adult resilience [42]. Future research could also benefit by examining the potential protective role of cognitive reappraisal against low self-esteem in cybervictimization, an important problem across adolescence [96].

### Implications for Treatments

The finding that cognitive reappraisal buffers the negative relationship between peer victimization and later self-esteem underscores the importance of enhancing victims’ ability for cognitive reappraising so as to reduce risk for low self-esteem over time. Cognitive behavioral therapy (CBT) could be promising in helping young victims reduce negative affect and replace distorted internal attributions of perceived mistreatment with more realistic and positive self-appraisals [10, 23, 88]. Drawing upon emotion socialization theory, intervention programs that aim to develop both parents’ and teachers’ ability to manage effectively emotions could also be beneficial in enhancing young victims’ engagement in cognitive reappraisal [20, 34]. Finally, emphasis should be given in promoting victims’ social skills so as to navigate healthy relationships with peers [28]. Social Skills Training Programs are shown to improve several domains of children’s functioning, including social interaction, sociometric status, and cognitive problems solving [27].

## Conclusion

To date, whether cognitive reappraisal buffers the negative relationship between peer victimization and later self-esteem across youth had not been explored. Using a longitudinal design the present study sought to address this gap by examining whether cognitive reappraisal moderated the longitudinal relationship between peer victimization and self-esteem in early adolescence. Results showed that peer victimization was negatively related to later self-esteem, but only for young individuals reporting low levels of cognitive reappraisal. Inversely, for young individuals reporting high levels of cognitive reappraisal, peer victimization was not found to predict any significant changes in self-esteem over time. Enhancing early adolescents’ cognitive reappraisal and social skills could be a promising avenue for preventing self-esteem erosion following peer victimization.

### Summary

Poor self-esteem associates closely to youth overall maladjustment and appears to be influenced by prior exposure to peer victimization experiences [67]. However, youth with poor self-esteem can also trigger peer harassment, and, thus, be trapped in a harmful vicious cycle that is hard to escape from [85]. Identifying factors that might protect young victims against self-esteem erosion over time could break this cycle and reduce risk for further abuse and later adjustment difficulties. Despite of evidence showing that cognitive reappraisal can reduce the negative relationship between peer victimization and later self-esteem in adolescence, this possibility had not been addressed in any published article yet. Drawing upon the Bi-Dimensional Framework for resilience [45] and extant research, the present study used a longitudinal two-wave design to examine the potential moderating role of cognitive reappraisal in the prospective relationship between peer victimization and self-esteem in 285 early adolescents (*M*_age_ = 10.53 years). To increase precision of findings the long-term impact of self-esteem on peer victimization was also tested. Peer victimization was found to be negatively related to later self-esteem, but only for early adolescents reporting low levels of cognitive reappraisal. For adolescents with high levels of cognitive reappraising, peer victimization was not found to predict any long-term changes in self-esteem. The impact of self-esteem on later peer victimization was not statistically supported. The present findings imply that it may not be peer victimization per se that deteriorates self-esteem, but rather the way young victims interpret the experience of being peer harassed [63]. Low self-esteem may be more likely to elicit peer victimization in later stages of human development when it becomes more stable and trait-like [17]. Enhancing early adolescents’ cognitive reappraisal could, therefore, be promising for reducing self-esteem erosion following peer victimization. Intervention programs should also focus on developing young victims’ social skills so as to help them function adaptively in the peer context. Based on emotion socialization theory, promoting parents’ and teachers’ ability to manage effectively emotions could also be helpful in increasing young victims’ use of cognitive reappraisal [20, 34].

## Data Availability

The datasets generated and/or analyzed during the current study are not publicly available but are available from the corresponding author on reasonable request.
